# Molecular mechanisms and applications of natural transformation in bacteria

**DOI:** 10.3389/fmicb.2025.1578813

**Published:** 2025-06-24

**Authors:** Changcheng Niu, Hao Wu, Xiaona Wang, Liying Hu, Yanping Han, Jianjun Qiao

**Affiliations:** ^1^School of Chemical Engineering and Technology, Tianjin University, Tianjin, China; ^2^Precision Nutrition and Health Research and Development Center, Zhejiang Research Institute of Tianjin University (Shaoxing), Shaoxing, China; ^3^Key Laboratory of Systems Bioengineering (Ministry of-Education), Tianjin University, Tianjin, China; ^4^State Key Laboratory of Synthetic Biology, Tianjin University, Tianjin, China

**Keywords:** natural transformation, competence, application, molecular mechanisms, activation, biological functions

## Abstract

Natural transformation is a process in which bacteria uptake exogenous DNA from the environment during a transient physiological state called competence. The DNA can either autonomously replicate or integrate into the bacterial chromosome through homologous recombination. Natural transformation has been studied for nearly a century. Recently, the rapid development of synthetic biology has led to the widespread use of natural transformation as a gene-editing tool for modifying industrial strains. A better understanding of the basic principles of natural transformation can enhance its biotechnological applications. This article provides a detailed overview of the natural transformation process, from initiation to completion. It focuses on the molecular mechanisms involved in natural transformation in both Gram-positive and Gram-negative bacteria. The article also analyzes factors that influence the activation of natural transformation, detailing the regulatory processes and signaling pathways involved. It further explores the potential biological functions of natural transformation. Finally, it discusses various applications of natural transformation in gene editing, offering insights into its potential for modifying industrial strains.

## 1 Introduction

Natural transformation is a process where bacteria take up exogenous DNA from the environment when they enter a transient physiological state known as competence. This DNA can either replicate autonomously, for instance as an episome or plasmid, or integrate into the chromosome through homologous recombination (Blokesch, 2016). The process of natural transformation was first discovered by Griffith in *Streptococcus pneumoniae* in 1928. The Gram-negative bacteria most frequently studied include *Vibrio cholerae*, *Neisseria gonorrhoeae*, and *Acinetobacter baumannii*. Among Gram-positive bacteria, *Bacillus subtilis* and *Streptococcus pneumoniae* have been the subject of the most in-depth studies (O’Connell et al., 2022).

The process of natural transformation comprises two key stages: the activation of competence and the occurrence of transformation. The activation stage involves the establishment of natural competence in the strain (Feng et al., 2023). This process typically involves the expression and regulation of competence-specific regulatory genes. For example, the *comX* (Mulder et al., 2017) gene plays a critical role in streptococci; in *Bacillus subtilis*, *comK* (Nijland et al., 2010) is essential; while in *Staphylococcus aureus*, both *sigH* and *comK1* (Feng et al., 2023) jointly activate natural transformation. The competence activation regulatory network enables the strain to convert environmental stimuli into regulatory signals that control the transcription of specific genes. When these competence-specific genes are induced under certain conditions, the strain transitions from its normal physiological state to a specialized state, allowing it to take up exogenous DNA and enhancing its ability to interact with such DNA. This transformation is referred to as the activation process of competence (Fontaine et al., 2015). In bacteria, natural transformation proceeds through four distinct steps. First, it is initiated by the binding of exogenous double-stranded DNA to macromolecular complexes on the cell surface (Damke et al., 2022). Second, during the uptake phase, the exogenous DNA is imported into the periplasm (the space between the cell wall and membrane in Gram-positive bacteria or between the outer and inner membranes in Gram-negative bacteria). Third, the incoming DNA is directed to the inner cell membrane and transported into the cytoplasm as single-stranded DNA. Finally, in the cytoplasm, the DNA is integrated into the chromosome through homologous recombination. A special case in natural transformation involves autonomously replicating elements, such as plasmids. Upon entering the cell, these elements bypass homologous recombination and initiate replication using a specific origin of replication (ori) encoded on the plasmid. Typically, replication occurs through mechanisms like rolling-circle replication, triggered by the binding of the ori to specific replication initiation proteins, enabling the plasmid to replicate independently within the host.

## 2 Molecular mechanisms underlying natural transformation

For clarity, this paper will use representative Gram-positive and Gram-negative bacteria as examples. The process of natural transformation will be divided into two parts: DNA uptake and transport, as well as DNA homologous recombination.

### 2.1 Natural transformation in gram-positive bacteria

Natural transformation, an essential mechanism for genetic exchange, plays a critical role in the life cycle of Gram-positive bacteria. This article uses the typical Gram-positive bacteria *Streptococcus pneumoniae* and *Bacillus subtilis* as examples to detail the molecular mechanisms underlying natural transformation in Gram-positive bacteria. At least 15 genes have been reported to participate in this process (O’Connell et al., 2022), which are summarized in Table 1.

**TABLE 1:** Genes involved in natural transformation of gram-positive bacteria.

Gene	Organisms	Function	References
*comGA*	*B. subtilis, S. pneumoniae, Lactococcus* spp., *S. thermophilus*	ATPase	(Briley et al., 2011; Di Giacomo et al., 2022)
*comGB*	*B. subtilis, S. pneumoniae, Lactococcus* spp., *S. thermophilus*	Transmembrane protein	(Di Giacomo et al., 2022; O’Connell et al., 2022)
*comGC*	*B. subtilis, S. pneumoniae, Lactococcus* spp., *S. thermophilus*	Main pilin protein	(Chen et al., 2006; Laurenceau et al., 2013)
*comGD*	*B. subtilis, S. pneumoniae, Lactococcus* spp., *S. thermophilus*	Pilin protein	(Chen et al., 2006; Mann et al., 2013; Balaban et al., 2014; Oliveira et al., 2021)
*comGE*	*B. subtilis, S. pneumoniae, Lactococcus* spp., *S. thermophilus*	Pilin protein	(Chen et al., 2006; Mann et al., 2013; Balaban et al., 2014; Oliveira et al., 2021)
*comGF*	*S. pneumoniae, Lactococcus* spp., *S. thermophilus*	Pilin protein	(Balaban et al., 2014; Oliveira et al., 2021)
*comGG*	*B. subtilis, S. pneumoniae, Lactococcus* spp., *S. thermophilus*	Pilin protein	(Chen et al., 2006; Mann et al., 2013; Balaban et al., 2014; Oliveira et al., 2021)
*comC*	*B. subtilis, Lactococcus* spp., *S. thermophilus*	Prepilin peptidase	(Chen et al., 2006)
*comEA*	*B. subtilis, S. pneumoniae, Lactococcus* spp., *S. thermophilus*	DNA-binding proteins	(Ahmed et al., 2022)
*comEC*	*B. subtilis, S. pneumoniae, Lactococcus* spp., *S. thermophilus*	Channel proteins	(Hahn et al., 2021)
*comFA*	*B. subtilis, S. pneumoniae, Lactococcus* spp., *S. thermophiles*	ATPase	(Morales-Bayuelo, 2016)
*endA/nucA*	*B. subtilis(nucA), S. pneumoniae, Lactococcus* spp., *S. thermophilus*	Nucleases	(O’Connell et al., 2022)
*ssbA/ssbB*	*B. subtilis, S. pneumoniae, Lactococcus* spp., *S. thermophilus*	ssDNA-binding proteins	(Yadav et al., 2012)
*dprA*	*B. subtilis, S. pneumoniae, Lactococcus* spp., *S. thermophilus*	ssDNA-binding proteins	(Yadav et al., 2013)
*recA*	*B. subtilis, S. pneumoniae, Lactococcus* spp., *S. thermophilus*	ssDNA-binding proteins	(Bergé et al., 2003; Yadav et al., 2014)

#### 2.1.1 DNA uptake and transport in gram-positive bacteria

**FIGURE 1:**
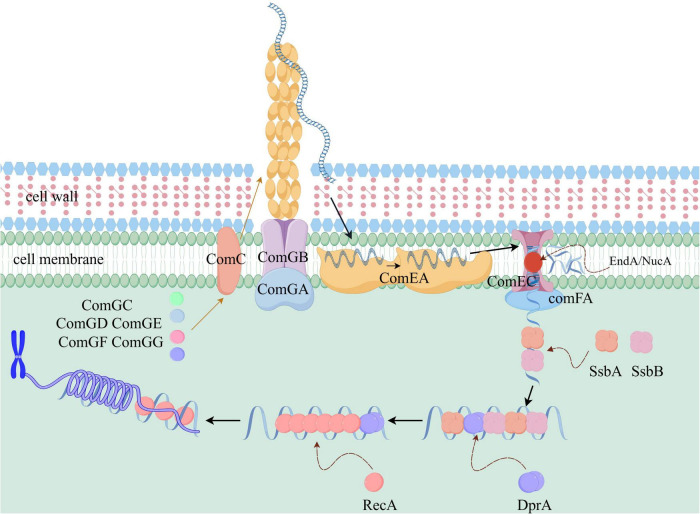
Schematic diagram of the molecular mechanism of natural transformation in *Streptococcus pneumoniae* (a Gram-positive bacterium). The pili, composed of ComGC and ComGD-GG, capture the exogenous DNA, with ComGA providing the energy. After pilus retraction, ComEA binds to the DNA, allowing it to diffuse across the outer membrane and be presented to ComEC. The EndA/NucA enzymes cleave the double-stranded DNA into single strands. Under the action of ComFA, which possesses translocase and ATPase activity, the single-stranded DNA enters the cell. SsbA and SsbB first bind to the single-stranded DNA to prevent degradation, followed by DprA, which loads RecA and stabilizes its binding. RecA searches for homologous regions and promotes recombination, thereby completing natural transformation.

As shown in Figure 1, the DNA uptake process is primarily facilitated by the *comG* operon, which contains seven genes: *comGA*, *comGB*, *comGC*, *comGD*, *comGE*, *comGF*, and *comGG*. Among these, *comGA* encodes a secreted ATPase, a member of the AAA + ATPase family, which provides energy for pilin protein movement during the uptake process. In *Bacillus subtilis*, the strain with the *comGA* gene deletion cannot bind to extracellular DNA, and the mutant also loses its natural transformation ability (Briley et al., 2011). *comGB* encodes a polymorphic transmembrane platform protein that supports the biosynthesis, export, and assembly of pilin proteins (O’Connell et al., 2022). *comGC* encodes the major pilin protein involved in bacterial pilus formation and exogenous DNA capture. It serves as the primary receptor for exogenous DNA and is essential for natural transformation. The morphology of this pilus has been described in the literature: it forms a long type IV pilus structure, 2–3 μM in length and 5 nm in width, extending from the bacterial surface (Laurenceau et al., 2013). Within the pilus, ComGC proteins are linked by covalent disulfide bonds (Chen et al., 2006). Additionally, *comGD* to *comGG* encode a group of minor pilin proteins, which, along with ComGC, form the bacterial pili. Notably, these minor pilin proteins (ComGD to ComGG) are also crucial for the DNA uptake process. Pili, composed of ComGC and ComGD-GF, adsorb environmental DNA through positively charged surface regions and pull the DNA toward the cell surface via contraction movement (Christman and Dalia, 2025). In *Streptococcus pneumoniae*, strains with deletions of the *comGD*, *comGE*, *comGF*, or *comGG* genes lose their natural transformation ability (Balaban et al., 2014). Furthermore, in *Streptococcus pneumoniae*, these four minor pilin proteins directly interact with each other. Specifically, ComGG stabilizes the minor pilin proteins ComGD and ComGF, and interacts with and stabilizes the major pilin protein, ComGC (Oliveira et al., 2021). In *Bacillus subtilis*, the minor pilin proteins ComGD, ComGE, and ComGG also interact directly with each other. Additionally, the processing of the minor pilin protein ComGG requires not only peptidases but also the involvement of other ComG proteins (Mann et al., 2013).

DNA transport requires the involvement of the *comC*, *comE*, and *comF* operons. *comC* encodes a precursor pilin processing peptidase essential for the post-translational modification and assembly of pilin proteins. ComC is also required for the translocation of the pilin protein ComGC to the outer membrane surface (Chen et al., 2006). *comEA* encodes a non-specific DNA-binding protein that stabilizes the binding of foreign DNA to the cell. ComEA helps internalized foreign DNA accumulate in the periplasm (Burghard-Schrod et al., 2022). In the absence of ComEA, DNA binding is reduced, and uptake is abolished completely (Hahn et al., 2021). Recent research shows that in *Bacillus subtilis*, an oligomerization domain within the ComEA protein structure promotes its polymerization. This oligomerization is critical in solution and DNA interactions, and is necessary for the transformation of Gram-positive bacteria (Ahmed et al., 2022). ComEA is a highly conserved DNA-binding protein. Its X-ray crystal structure reveals a helix-turn-helix (HTH) domain that directly interacts with double-stranded DNA. The positively charged surface of ComEA binds to the DNA phosphate backbone, forming a stable complex that facilitates DNA passage through the peptidoglycan layer into the cytoplasm (Ahmed et al., 2022; Foster et al., 2022). *comEC* encodes a membrane channel protein in the cytoplasmic membrane that forms a transport channel for foreign DNA (Pimentel and Zhang, 2018; Hahn et al., 2021). The structure of ComEC includes a channel formed by a transmembrane α-helix, which facilitates the transport of single-stranded DNA (ssDNA). It interacts with the negatively charged DNA phosphate backbone through positively charged amino acid residues, such as arginine and lysine, promoting DNA binding (Pimentel and Zhang, 2018; Christman and Dalia, 2025). The *comEB* gene in the *comE* operon plays a key role in localizing DNA uptake mechanisms to the cell poles. *comEB* encodes a deoxycytidylate deaminase, localized at the cell poles during natural transformation, influencing the localization of the ComGA protein and mediating polar localization. Burghard-Schrod et al. (2020) found that *comEB* is not essential for transformation, but its absence significantly reduces transformation frequency.

*comFA* encodes an ATPase containing a helicase-like domain. It hydrolyzes ATP through its ATP-binding cassette domain to supply the energy needed for transmembrane DNA transport. Studies show that the ComFA protein exhibits ssDNA-dependent ATPase activity, specifically stimulated by ssDNA. This supports the hypothesis that ComFA acts as a DNA uptake motor in Gram-positive bacteria (Morales-Bayuelo, 2016). Experiments have shown that ComFA exhibits ATP-dependent DNA translocase activity and can unidirectionally move along single-stranded DNA at a rate of approximately 10 bp/s, providing the driving force for DNA translocation across the membrane. Its helicase activity may help unwind double-stranded DNA to facilitate the uptake of single-stranded DNA (Foster et al., 2022). The *comFC* gene encodes a membrane-associated protein, shown to play a crucial role in natural transformation. Specifically, it promotes the transport of foreign DNA and facilitates homologous DNA recombination in the cytoplasm (Damke et al., 2022). Additionally, nucleases EndA/NucA are involved in the DNA uptake process. These enzymes bind to the ComEC protein, cleaving foreign double-stranded DNA into single-stranded DNA and releasing soluble nucleotides into the environment (O’Connell et al., 2022).

In summary, DNA uptake and transport in Gram-positive bacteria occurs as follows: First, exogenous DNA from the environment is captured by bacterial pili, primarily composed of the ComGC protein, with secondary involvement of the ComGD-GG protein. The ComGA protein provides the energy required for pilus movement. At this stage, the binding of exogenous DNA to the pili is weak. In the next step, the pilus retracts, drawing the DNA to the cell surface. The DNA-binding protein ComEA then interacts with and binds the exogenous DNA. Under the influence of ComEA, the DNA diffuses freely within the outer membrane. The exogenous DNA is subsequently delivered to the membrane channel protein ComEC on the cytoplasmic membrane. On ComEC, the exogenous double-stranded DNA is cleaved into single strands by the EndA/NucA enzymes. Single-stranded DNA is then translocated into the cell through the translocase activity of the ComFA protein. Meanwhile, soluble nucleotides are released into the surrounding environment, and the ATPase activity of ComFA provides the energy necessary for this process.

#### 2.1.2 Homologous recombination of DNA in gram-positive bacteria

DNA homologous recombination during bacterial natural transformation is a complex and tightly regulated process requiring the coordinated involvement of several ssDNA-binding proteins, including DprA, SsbB, SsbA, and RecA (Di Giacomo et al., 2022). As shown in Figure 1, SsbA and SsbB are expressed in most bacteria with a cellular recombination mechanism. They typically exist as tetramers in the cell, binding to ssDNA in this form. Both proteins can form mixed complexes on ssDNA, though heterotetramer formation has not been detected (Yadav et al., 2012). DprA plays a key role in bacterial natural transformation (Serrano et al., 2018). DprA consists of two domains: the N-terminal OB-fold domain, which binds single-stranded DNA, and the C-terminal RecA interaction domain. Through a cooperative binding mechanism, DprA first binds to single-stranded DNA and then recruits RecA to form a nucleoprotein filament, facilitating homologous recombination (Sharma et al., 2023). During this process, DprA receives incoming ssDNA and promotes the exchange of SsbA and SsbB proteins on the ssDNA (Yadav et al., 2013). DprA also assists in loading RecA to facilitate homologous-directed chromosomal transformation and DNA strand annealing (Sharma et al., 2023). The dimeric form of DprA is crucial for optimal ssDNA binding. RecA’s main function in natural transformation is to form helical nucleoprotein filaments on ssDNA and catalyze the integration of homologous ssDNA into the competent cell genome (Bergé et al., 2003; Yadav et al., 2014). On the cytoplasmic side of the membrane, ComFA forms a complex with ComFC and DprA, protecting ssDNA from degradation by intracellular nucleases (Diallo et al., 2017; Le et al., 2017).

In summary, homologous recombination in Gram-positive bacteria proceeds as follows: SsbA and SsbB, due to their much higher affinity for single-stranded DNA compared to DprA or RecA, are the first to bind the incoming DNA, fully coating it to protect it from degradation by intracellular nucleases (Le et al., 2017). The homologous recombination mediator protein DprA then loads the RecA enzyme onto the single-stranded DNA, playing a critical role. The DprA dimer further stabilizes RecA binding to the single-stranded DNA. RecA then forms a helical nucleoprotein filament on the single-stranded DNA, stretching it 1.5 times its natural length to search for sequence homology with the bacterial chromosome. Once sufficient homology is found, base pairing occurs, promoting recombination of thousands of base pairs and protecting the DNA from degradation. This catalyzes the integration of homologous DNA into the genome of the competent cell, completing natural transformation.

### 2.2 Gram-negative bacteria

*Acinetobacter baylyi* ADP-1 is one of the earliest identified representative strains for natural transformation in Gram-negative bacteria (Hülter et al., 2017). This article uses *Acinetobacter baylyi* as an example to provide a detailed overview of the molecular mechanisms of natural transformation in Gram-negative bacteria. At least 16 genes are reported to be involved in the natural transformation process of *A. baylyi* (Averhoff et al., 2021), as summarized in Table 2.

**TABLE 2:** Essential genes for natural transformation in gram-negative bacteria.

Gene	Organisms	Function	References
*comP*	*A. baylyi*	Main pilin protein	(Porstendörfer et al., 1997)
*comB*	*A. baylyi*	Pilin protein	(Herzberg et al., 2000)
*comE*	*A. baylyi*	Pilin protein	(Busch et al., 1999)
*comF*	*A. baylyi*	Pilin protein	(Busch et al., 1999)
*pilV*	*A. baylyi*	Pilin protein	(Averhoff et al., 2021)
*pilX*	*A. baylyi*	Pilin protein	(Averhoff et al., 2021)
*fimT*	*A. baylyi*	Pilin protein	(Averhoff et al., 2021)
*comC*	*A. baylyi*	Adhesin	(Morand et al., 2009; Giltner et al., 2012)
*comQ*	*A. baylyi*	Channel proteins	(Majewski et al., 2018; Silva et al., 2020)
*comEA*	*A. baylyi*	DNA-binding proteins	(Ahmed et al., 2022)
*comA*	*A. baylyi*	Channel proteins	(Friedrich et al., 2001)
*pilB*	*A. baylyi*	Pilus assembly ATPase	(Leong et al., 2017)
*pilT/pilU*	*A. baylyi*	Pilus retraction ATPase	(Leong et al., 2017)
*ssbA/ssbB*	*A. baylyi*	ssDNA-binding proteins	(Averhoff et al., 2021)
*dprA*	*A. baylyi*	ssDNA-binding proteins	(Yadav et al., 2013; Averhoff et al., 2021)
*recA*	*A. baylyi*	ssDNA-binding proteins	(Yadav et al., 2014; Averhoff et al., 2021)

#### 2.2.1 DNA uptake and transport in gram-negative bacteria

**FIGURE 2:**
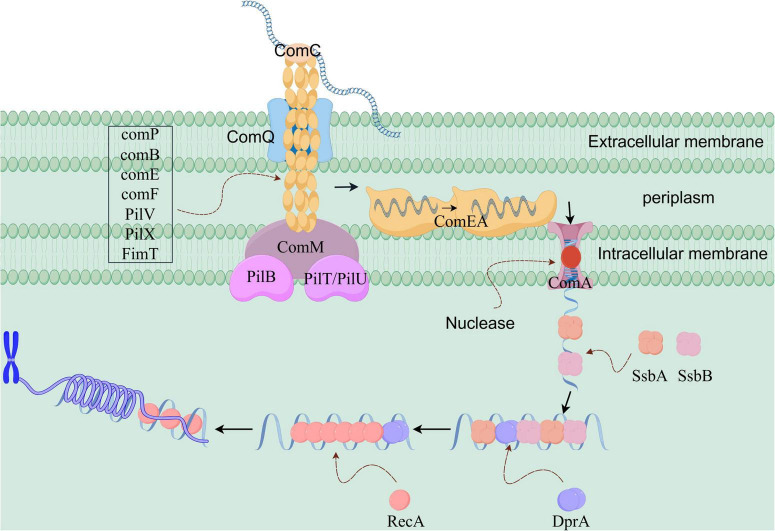
Schematic diagram of the molecular mechanism of natural transformation in Acinetobacter baylyi (a Gram-negative bacterium). The pili first recognize and bind to the exogenous DNA. The energy provided by PilT/PilU drives pilus retraction, passing through the ComQ channel and pulling the DNA into the periplasm. After binding to ComEA, the DNA is transported to the ComA channel protein with its assistance. Under the action of an unknown helicase, the DNA becomes single-stranded as it enters the bacterium. SsbA and SsbB bind to the single-stranded DNA, protecting it and guiding it to DprA. DprA aids in loading RecA onto the single-stranded DNA, promoting its integration into the chromosome.

As shown in Figure 2, in Gram-negative bacteria, the DNA uptake process during natural transformation is primarily mediated by Type IV pili (T4P) (Leong et al., 2017). T4P assemble into a helical fiber structure via non-covalent bonds, anchoring in the inner membrane and spanning across the periplasm to the outer membrane. The major pilin proteins, including ComP, ComB, PilV, PilX, and FimT, along with the accessory pilin proteins ComE and ComF, collectively form the pili in *Acinetobacter baylyi* (Averhoff et al., 2021). T4P are responsible for recognizing and internalizing foreign DNA. Bacteria generally recognize DNA from any source, although exceptions exist, such as Neisseria species, which only recognize DNA containing a DNA uptake sequence (DUS) (Maughan et al., 2010).

The *comP* gene was the first identified natural transformation-related gene in Acinetobacter, and its essential role in DNA uptake was demonstrated by Porstendörfer et al. (1997). They also discovered that the glycosylated form of ComP is a common feature of extracellular proteins. Localization experiments have shown that ComP is present on the bacterial outer membrane, periplasm, and inner membrane (Porstendörfer et al., 2000). In addition to *comP*, several other genes contribute to the formation of T4P, such as *comB* (Herzberg et al., 2000), *comE*, and *comF* (Busch et al., 1999). Notably, the pilin subunits that make up the pili can vary among bacterial species.

In Gram-negative bacteria, natural transformation may also involve ComC (pilC), an adhesin that mediates attachment to biological surfaces (Giltner et al., 2012). Due to its role in surface adhesion, ComC is typically predicted to localize at the tip of the pilus (Morand et al., 2009). Link et al. (1998) demonstrated experimentally that ComC is essential for DNA binding and uptake. Type IV pili bind directly to double-stranded DNA through tip-specific proteins, relying on electrostatic interactions, such as those involving positively charged lysine residues. This binding exhibits sequence preference, particularly for DUS sequences. Additionally, during DNA uptake in Gram-negative bacteria, the ComQ protein (PilQ) is involved. PilQ is a secretion-associated protein anchored in the outer membrane (Silva et al., 2020). Multiple ComQ monomers form a polymeric ring structure in the outer membrane, acting as a gateway for foreign DNA entry into the cell (Approximately 10 nm in diameter) (Majewski et al., 2018). The polymeric structure carries out its transmembrane transport function through a β-sheet barrel conformation (Ellison et al., 2018). The ATPases PilB and PilT/PilU mediate the extension and retraction of the pili (Chiang et al., 2008; Leong et al., 2017). After pilus retraction, the DNA-binding protein ComEA binds to the internalized foreign DNA. In this case, ComEA, a periplasmic DNA-binding protein, has its N-terminal domain interact electrostatically with the DNA phosphate backbone through positively charged amino acid residues, such as arginine and lysine clusters, while its C-terminal transmembrane domain anchors the DNA to the inner membrane (Dubnau and Blokesch, 2019; Ahmed et al., 2022). Additionally, ComA is involved in natural transformation; it is anchored in the inner membrane and forms a channel that transports foreign DNA across the inner membrane (Averhoff et al., 2021; Friedrich et al., 2001). Notably, similar to Gram-positive bacteria, a DNA helicase likely acts on the ComA protein to unwind double-stranded DNA. However, the identity of this helicase remains unknown.

In summary, the DNA uptake and transport mechanism in Gram-negative bacteria proceeds as follows: When DNA is present in the external environment and recognized by pili, the pili, composed of various pilin subunits, initially bind to the exogenous DNA. At this stage, the binding between the DNA and the bacteria is weak. The pili then retract, a process powered by energy from PilT/PilU. During retraction, the pili pass through the ComQ protein channel, drawing the exogenous DNA into the periplasm. Meanwhile, the exogenous DNA binds to the DNA-binding protein comEA, facilitating its storage and accumulation at the cell membrane. Subsequently, with the help of ComEA, the exogenous DNA moves freely and eventually reaches the ComA channel protein. With the aid of an unidentified DNA helicase, the DNA is converted into single-stranded form and enters the bacterial interior through the channel protein, completing the DNA uptake process.

#### 2.2.2 Homologous recombination of DNA in gram-negative bacteria

In Gram-negative bacteria, the process of DNA homologous recombination is similar to that in Gram-positive bacteria and will not be discussed further.

## 3 Activation of natural transformation

### 3.1 Factors influencing the activation of natural transformation

The activation of a strain’s natural transformation ability has been a major focus of research in microbiology. Numerous studies have identified various factors that play key roles in this complex biological process. This chapter will explore these factors in three areas: the growth state of the strain, quorum sensing, and environmental stress (Friedrich et al., 2001).

#### 3.1.1 Growth state of the strain

The growth state of the strain is the most important factor in activating its natural transformation ability. For example, in the Gram-positive bacterium *Streptococcus pneumoniae*, natural transformation ability is rapidly and simultaneously activated in nearly all cells during the exponential growth phase, lasting for about 15 min before declining sharply at a similar rate (Claverys et al., 2006). In contrast, in *Bacillus subtilis*, a Gram-positive bacterium, natural transformation ability is activated during the stationary phase of growth (Hamoen et al., 2003), with only about 10% of the cells exhibiting this activation (Maamar and Dubnau, 2005). While the activation of natural transformation typically occurs at specific growth stages for most bacteria, some strains, such as *Neisseria gonorrhoeae* (Callaghan and Dillard, 2019), retain the ability to undergo natural transformation regardless of their growth state.

#### 3.1.2 Quorum sensing

Quorum sensing also plays a critical role in activating natural transformation ability. Quorum sensing is a mechanism by which bacteria coordinate group behaviors through the secretion and detection of signaling molecules (Moreno-Gámez et al., 2017). These signaling molecules are essential for activating a strain’s natural transformation ability. When the bacterial population density reaches a threshold, the concentration of signaling molecules increases, activating natural transformation. A well-known example is *Bacillus subtilis* and *Streptococcus pneumoniae*, both of which rely on this mechanism to activate natural transformation.

#### 3.1.3 Environmental stress

##### 3.1.3.1 Antibiotic stress and DNA damage

Prudhomme et al. (2006) first demonstrated that antibiotic stress and DNA damage can induce natural transformation in *Streptococcus pneumoniae* (Prudhomme et al., 2006). Building on this, Slager et al. (2014) experimentally showed that mitomycinC or norfloxacin can activate natural transformation in *S. pneumoniae*. The mechanism involves antibiotics targeting bacterial DNA replication, leading to replication fork stalling. Meanwhile, DNA replication continues, resulting in an increased copy number of genes near the initiation sites. Since the genes required for natural transformation (*comAB* and *comCDE*) are located in this region, their activation triggers the process (Slager et al., 2014). Stevens et al. (2011) also showed that aminoglycosides (e.g., streptomycin and kanamycin) can activate natural transformation in *S. pneumoniae*. These antibiotics cause misfolded proteins to accumulate, which are then bound by the membrane-associated serine protease (HtrA) instead of the quorum sensing peptide (CSP). This leads to the extracellular accumulation of CSP, thereby activating competence (Stevens et al., 2011). Literature also reports that ampicillin and clavulanic acid can activate natural transformation. The mechanism involves these compounds targeting specific penicillin-binding proteins, leading to their loss and the formation of chains in *S. pneumoniae*. CSP remains trapped in these chain-forming cells, increasing the CSP concentration within the cells and thus activating natural transformation (Sturød et al., 2018). However, not all antibiotics exert this effect. Antibiotics like erythromycin, tetracycline, neomycin, carbapenems, cephalosporins, rifampin, vancomycin, and ampicillin do not activate natural transformation in *S. pneumoniae*. This indicates that the activation or inhibition of competence in *S. pneumoniae* depends on the type of antibiotic used (Huang et al., 2021).

##### 3.1.3.2 Chitin

Several studies have shown that certain members of the *Vibrionaceae* family, including *Vibrio cholerae* (Meibom et al., 2005), *Vibrio fischeri* (Pollack-Berti et al., 2010), *Vibrio vulnificus* (Gulig et al., 2009), and *Vibrio parahaemolyticus* (Chen et al., 2010), can induce natural transformation in the presence of chitin. For example, *Vibrio parahaemolyticus* can degrade insoluble chitin with the help of chitinases, producing soluble N-acetylglucosamine oligosaccharides (GlcNAcn), which activate natural transformation. The activation mechanism involves the following: First, the chitin-responsive regulatory protein CytR controls the expression of intracellular and periplasmic chitinases, regulating the release of GlcNAc6 from insoluble chitin and its conversion into smaller GlcNAc residues in the periplasm. These GlcNAc residues (β-GlcNAc6) upregulate competence-related genes, including *pilA*, *pilB*, *comEA*, and *qstR*. Meanwhile, the transformation-promoting factor X protein, TfoX, activates natural transformation in a chitin-dependent manner through CytR. Additionally, the quorum-sensing regulatory protein OpaR indirectly influences natural transformation by regulating the extracellular nuclease Dns. In summary, chitin induces natural transformation in *Vibrio parahaemolyticus* through a series of enzymes and regulatory factors (Debnath and Miyoshi, 2022).

##### 3.1.3.3 Nutrients

Rich nutrient environments can sometimes influence a strain’s ability to undergo natural transformation. Experiments have shown that *Acinetobacter calcoaceticus* in soil, where transformation is undetectable, can be easily induced to undergo chromosomal DNA natural transformation when nutrients are available. Under starvation conditions, cells may activate a series of stress response mechanisms to survive and adapt, which can, in turn, influence their natural transformation ability. For example, yeast cells require nutrient starvation to undergo transformation, as natural transformation does not occur in standard growth media (Nevoigt et al., 2000). Furthermore, Sinha et al. (2013) demonstrated that the absence of purine nucleotides triggers the activation of natural transformation in *Haemophilus influenzae*. The mechanism involves the consumption of extracellular purine nucleotides, which activates the expression of the CRP-dependent regulatory factor Sxy, thereby inducing natural transformation in *H. influenzae*. High concentrations of purine nucleotides reduce Sxy translation, thereby decreasing the bacterium’s natural transformation ability. This inhibitory effect occurs by repressing Sxy translation, rather than altering the mRNA expression of Sxy (Sinha et al., 2013). Similar mechanisms have also been reported in *Escherichia coli* and *Vibrio cholerae* (Antonova et al., 2012).

##### 3.1.3.4 Artificial sweeteners

Yu et al. (2022) demonstrated that the Gram-positive bacterium *Bacillus subtilis* exhibited increased cell membrane permeability and activated natural transformation under the influence of four commonly used artificial sweeteners: saccharin, sucralose, aspartame, and acesulfame potassium. This led to the upregulation of genes involved in DNA uptake and transport (com genes) (Yu et al., 2022).

In addition to the previously mentioned environmental stresses—such as antibiotic stress, DNA damage, chitin, nutrient richness, starvation, and artificial sweeteners—that induce bacterial natural transformation, other factors can also influence this ability. For example, bacteria such as *Streptococcus pneumoniae* and *Bacillus subtilis* activate natural transformation under specific environmental stresses (Claverys et al., 2006). Similarly, *Legionella pneumophila* can activate its natural transformation ability under genotoxic stress conditions, particularly when exposed to ultraviolet radiation (Charpentier et al., 2011).

### 3.2 Molecular mechanisms of natural transformation activation

During genetic exchange via natural transformation, bacteria exhibit remarkably diverse and precise molecular mechanisms to activate competence. Different bacterial groups have evolved specialized signaling systems that regulate key transcription factors, integrate environmental signals, and sense cell density to initiate the expression of genes involved in competence development and DNA uptake. This article introduces three representative molecular mechanisms: the ComAB ABC transporter and ComCDE three-component regulatory system in *Streptococcus pneumoniae*, the ComRS signaling system in *Streptococcus thermophilus*, and the ComK signaling pathway in *Bacillus subtilis*.

#### 3.2.1 The ComCDE three-component regulatory system in *Streptococcus pneumoniae*

**FIGURE 3:**
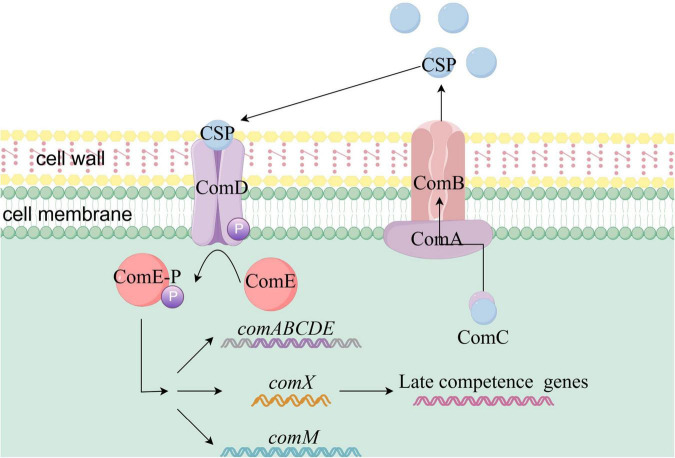
Schematic diagram of ComCDE-mediated activation of natural competence in *Streptococcus pneumoniae.* The quorum-sensing molecule CSP is derived from the ComC precursor protein, which is transported by ComAB and processed by ComA. CSP binds to membrane-anchored ComD, triggering its autophosphorylation. The phosphate group is transferred to ComE, initiating its phosphorylation and activating genes such as *comABCDE*, *comX*, and *comM*. ComX activates late competence genes through interaction with RNA polymerase. Phosphorylation of ComE results in the formation of a positive feedback loop for comABCDE. External stimuli promote CSP accumulation, activating this system.

The ComCDE competence activation system in *Streptococcus pneumoniae* is illustrated in Figure 3. ComD is a histidine kinase dimer anchored in the cell membrane, acting as a receptor for the quorum sensing molecule (CSP) and sensing CSP concentration. Upon detecting a threshold concentration of CSP, ComD undergoes autophosphorylation. CSP is a 17-amino acid secreted signaling peptide (Aggarwal et al., 2020), processed from the precursor protein ComC. The ABC transporter ComAB, also embedded in the cell membrane, is responsible for CSP secretion. During secretion, the protease domain of ComA cleaves the N-terminal signal peptide of ComC at a unique Gly-Gly motif, producing the mature form of CSP (Håvarstein et al., 1995). Most *S. pneumoniae* strains produce two variants of the quorum sensing peptide, CSP-1 and CSP-2, which correspond to two variants of the ComD proteins, ComD1 and ComD2. The corresponding CSPs more effectively activate natural transformation in strains expressing the corresponding ComD receptors (Milly et al., 2023).

Yang et al. (2017) experimentally demonstrated that CSP-1 and CSP-2 can induce competence in strains expressing the ComD2 and ComD1 receptors, respectively. However, due to reduced binding affinity, the efficacy of these peptides decreased by 40-fold (Yang et al., 2017). ComE is a homologous response regulator of ComD. After ComD undergoes phosphorylation, it transfers its phosphoryl group to a conserved aspartic acid residue in ComE, initiating the phosphorylation of ComE (Martin et al., 2013). Phosphorylated ComE becomes active and can recognize and activate the transcription of approximately 20 early competence genes, including *comABCDE* (Martin et al., 2013), the natural transformation regulator *comX* (Peterson et al., 2004), and the immune protein gene *comM*. The ComX, in association with RNA polymerase, forms a holoenzyme that recognizes a specific DNA sequence known as the com-box. This activation induces the transcription of over 100 late competence genes involved in DNA uptake and homologous recombination, fully activating natural transformation (Luo and Morrison, 2003).

Simultaneously, phosphorylation of ComE establishes a positive feedback loop for the comABCDE system. CSP accumulates extracellularly, triggering ComE phosphorylation, which further activates the expression of *comABCDE* and accelerates CSP accumulation outside the cell (Martin et al., 2013). This feedback loop continues to activate bacterial natural transformation. Thus, when bacteria are exposed to environmental stimuli, such as antibiotic stress, CSP accumulates in the bacterial environment, activating the comCDE regulatory system and triggering natural transformation.

#### 3.2.2 The ComRS signaling system activating natural transformation in *Streptococcus thermophilus*

**FIGURE 4:**
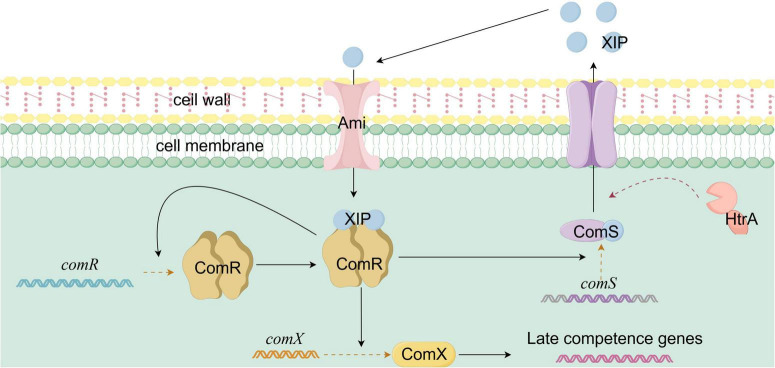
Schematic diagram of ComRS-mediated natural competence activation in Streptococcus thermophilus. The ComRS system of Streptococcus thermophilus consists of the transcriptional regulator ComR and the precursor signal peptide ComS. In its basal state, ComS exists as a precursor, which, upon activation, is processed by proteases into the mature pheromone peptide XIP. XIP is secreted extracellularly and accumulates. Once its concentration reaches a threshold, it enters the cell through oligopeptide permeases. XIP binds to ComR, inducing a conformational change. Two molecules of XIP and two molecules of ComR form a heterotetramer, which binds to the comX gene promoter, activating its expression. ComX directs RNA polymerase to initiate the transcription of transformation-related genes. Meanwhile, the ComR-XIP complex promotes the expression of the comS and comR genes, forming a positive feedback loop.

The ComRS system in *Streptococcus thermophilus* serves as the central regulatory mechanism for natural transformation, integrating quorum sensing and environmental signals, with the specific mechanism shown in Figure 4. The system comprises the cytoplasmic transcriptional regulator ComR and the precursor signaling peptide ComS (Knoops et al., 2022b). Under basal conditions, ComS is produced as an inactive precursor. Upon competence activation, proteases such as HtrA process ComS into a mature signaling peptide, XIP (7–9 amino acids in length). Traditionally, XIP was thought to be secreted into the extracellular environment, accumulating in response to cell density and environmental conditions (Knoops et al., 2022a,b). ComR, a member of the RNPP family of transcriptional regulators, contains an N-terminal peptide-binding pocket and a C-terminal helix-turn-helix (HTH) DNA-binding domain. Upon binding XIP, ComR undergoes a conformational change, enabling it to bind DNA (Talagas et al., 2016). The conserved and variable regions of the peptide-binding pocket mediate interaction with XIP’s core structure and species-specific recognition, respectively (Shanker et al., 2016). In *S. thermophilus*, ComR specifically recognizes XIP through residues W35 (tryptophan) and E102 (glutamic acid) (Talagas et al., 2016). When extracellular XIP reaches a threshold concentration, it re-enters the cell through oligopeptide permeases like Ami. Inside the cell, XIP binds ComR, inducing conformational changes. Two XIP molecules and two ComR molecules form a heterotetrameric complex that binds the *comX* promoter region (comR-box) to activate *comX* expression (Knoops et al., 2022b). ComX guides RNA polymerase to bind the promoters of transformation-related genes, triggering the transcription of genes responsible for DNA uptake and recombination. Simultaneously, the ComR-XIP complex enhances the transcription of *comS* and *comR*, forming a positive feedback loop that amplifies the activation of natural transformation. Recent studies have revealed an alternative “intracellular activation pathway,” where unprocessed ComS binds ComR directly, bypassing secretion and reuptake (Underhill et al., 2018). Besides cell density, the ComRS system integrates various environmental cues. For instance, under nutrient-limiting conditions like carbon source restriction, metabolic intermediates may indirectly regulate *comS* expression or affect XIP stability (Knoops et al., 2022b).

#### 3.2.3 The ComK signaling system activating natural transformation in *Bacillus subtilis*

**FIGURE 5:**
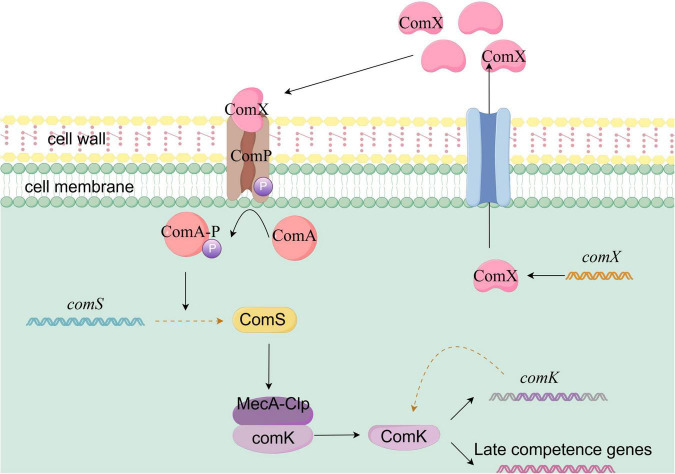
Schematic diagram of ComK-mediated natural competence activation in *Bacillus subtilis.* In *Bacillus subtilis*, natural competence activation is primarily controlled by the ComK system. As cell density increases, the quorum sensing signal molecule ComX accumulates to a threshold level and is recognized by the membrane receptor ComP. This triggers ComP autophosphorylation and the subsequent phosphorylation of ComA (ComA∼P), which activates transcription of the *comS* gene. The ComS protein inhibits the MecA-ClpC protease complex, preventing the degradation of ComK and promoting its accumulation. Once ComK reaches a threshold concentration, it enhances its own transcription through a positive feedback loop. The quorum sensing signal, via ComS regulation of ComK stability, synergizes with ComK’s own transcriptional positive feedback mechanism to ultimately drive *Bacillus subtilis* into a state of natural competence.

The natural transformation ability of *Bacillus subtilis* is primarily regulated by the ComK system, which involves multiple layers of signal sensing, transcriptional regulation, and protein cooperation. This process precisely controls the expression of transformation-related genes through a synergy between quorum sensing signals and transcriptional auto-regulation, as illustrated in Figure 5. ComK serves as the core transcriptional activator for natural transformation. Its activation follows an all-or-nothing cooperative binding mechanism. When ComK concentration reaches a threshold, it promotes its own transcription through a positive feedback loop, creating an “all-or-nothing” gene expression switch. This self-amplifying feedback mechanism is crucial for the cell-specific activation of natural transformation within a population (Dogsa et al., 2021; Rosenblum et al., 2021)

The quorum sensing system ComQXPA plays a central role in ComK activation. As the cell density of *Bacillus subtilis* increases, ComX, a modified peptide encoded by the comX gene, accumulates extracellularly as a quorum sensing signal molecule. When its concentration reaches a threshold, ComX is recognized by the membrane receptor histidine kinase ComP, leading to ComP’s self-phosphorylation. The phosphate group is then transferred to the transcription factor ComA (Dogsa et al., 2021). Phosphorylated ComA (ComA∼P) subsequently activates the transcription of the *comS* gene. The ComS protein inhibits the ClpCP protease, preventing the degradation of ComK and promoting its accumulation within the cell (Lu et al., 2023).

In non-competent cells, comK transcription is repressed by several global regulatory factors, such as *Spo0A* and *CodY*. A small amount of ComK produced is sequestered by the MecA-ClpC protease complex, ensuring that ComK remains at low levels (Stiegelmeyer and Giddings, 2013). Another critical aspect of ComK activation is the regulation of its protein stability. The MecA-ClpC protease complex typically degrades ComK to maintain its low steady-state level in non-competent cells. However, when ComS binds to MecA-ClpC, it blocks the protease’s degradation of ComK, allowing ComK to accumulate and function. The quorum sensing signal, by regulating ComK stability through ComS, works together with ComK’s own transcriptional positive feedback mechanism, ultimately driving *Bacillus subtilis* into a natural transformation state.

## 4 Biological functions of natural transformation

Natural transformation is a remarkable biological process that plays a vital role in inheritance and evolution, significantly influencing the survival and development of organisms. Despite its importance, the precise biological functions of natural transformation remain a subject of ongoing debate. This chapter aims to explore the potential biological functions of natural transformation, as discussed in the current literature.

### 4.1 Promoting genome evolution and environmental adaptation in strains

Natural transformation plays a crucial role in the genomic evolution and environmental adaptability of bacterial strains. Experiments by Engelmoer et al. (2013) showed that under selective pressure, *Streptococcus pneumoniae* maintains genomic DNA stability through its natural transformation ability. This process helps reduce non-synonymous mutations, enhancing adaptability in stressful environments (Engelmoer et al., 2013). Additionally, when natural transformation involves genes encoding harmful traits, it aids in their removal, promoting genomic evolution (de Visser and Elena, 2007). *Thermus thermophilus* HB27 thrives in extreme environments, with temperatures up to 85°C, thanks to its highly efficient DNA uptake system and robust natural transformation ability (Schwarzenlander and Averhoff, 2006). However, while natural transformation can promote genomic evolution and adaptability, it often has detrimental effects on cells. If the genetic material introduced is harmful, it can lead to cellular damage. Some argue that exogenous DNA acquired through natural transformation is rarely beneficial, as it often comes from organisms that failed to survive under selective pressure (Blokesch, 2017).

### 4.2 Facilitate the dissemination of antibiotic resistance genes and virulence genes

Natural transformation facilitates the spread of antibiotic resistance genes. Antibiotic resistance in pathogenic bacteria has become a major global health threat, significantly reducing the efficacy of treatments for bacterial infections. *Campylobacter jejuni*, a major foodborne pathogen, has progressively enhanced its antibiotic resistance through natural transformation and uptake of exogenous DNA (Bae et al., 2014). As a result, the effectiveness of antibiotics in treating related diseases has been significantly reduced, posing a serious threat to human health. Natural transformation also facilitates the transfer of virulence genes, increasing the pathogenicity of bacteria. Strains with strong virulence genes can cause more severe diseases, posing a greater threat to human and animal health. Rasko et al. (2011) found that the *Escherichia coli* strain responsible for the hemolytic uremic syndrome outbreak in Germany resulted from natural transformation.

### 4.3 Facilitate genomic repair

Natural transformation facilitates the repair of genomic DNA in bacterial strains. As mentioned previously, antibiotic stress and DNA damage can induce natural transformation (Prudhomme et al., 2006). As early as 1991, a study on *Bacillus subtilis* reported that exogenous DNA taken up through natural transformation can serve as a template for recombinational repair of genomic damage. All organisms possess the ability to repair abnormal DNA structures, and *Escherichia coli* serves as a model organism for studying microbial DNA repair. However, DNA repair genes vary significantly across bacterial species. Tønjum et al. (2004) demonstrated that homologous recombination through genes involved in natural transformation is a key DNA repair pathway (Tønjum et al., 2004).

### 4.4 Serve as a nutrient source

Natural transformation enables starving cells to use extracellular DNA as a nutrient source. As mentioned previously, some bacteria activate their natural transformation ability under nutrient-limiting conditions (Sinha et al., 2013). Cells relieve hunger stress by taking up DNA, as *de novo* nucleotide biosynthesis is costly. Previous studies have shown that *Escherichia coli* utilizes the DNA uptake mechanism in natural transformation to survive on extracellular DNA as the sole carbon and energy source. Although the direct link to natural transformation is unclear, certain *com* genes are essential for this function. In a minimal medium with inorganic salts, vitamin B1, and purified *E. coli* chromosomal DNA as the sole carbon and energy source, the growth of wild-type *E. coli* was more than 50 times higher than that of the YhiR mutant, demonstrating its ability to utilize exogenous DNA as a carbon source (Finkel and Kolter, 2001). Literature also reports that when purified salmon sperm DNA was added to M63 minimal medium, wild-type cell density increased by approximately 120 times compared to eight other *com* gene homolog mutant strains, further demonstrating *E. coli*’s ability to survive on salmon sperm DNA as the sole carbon and energy source (Palchevskiy and Finkel, 2006). Notably, only double-stranded DNA substrates (linear or circular) can serve as carbon and energy sources. *Escherichia coli* can efficiently catabolize double-stranded DNA as small as 24 bp, but cannot utilize single-stranded DNA or RNA as nutrients.

### 4.5 Participate in the formation and development of biofilms

Natural transformation also contributes to biofilm formation and development by taking up extracellular DNA to provide phosphates. Studies have shown that under phosphate deficiency, *Vibrio cholerae* induces the expression of extracellular nucleases, which degrade exogenous DNA taken up through natural transformation, contributing to biofilm formation. Extracellular DNA, a key component of *Vibrio cholerae* biofilms, plays a role in processes such as formation, detachment, nutrient acquisition, and colonization adaptability, in conjunction with extracellular nucleases (Seper et al., 2011).

## 5 Practical applications of natural transformation

Natural transformation, a natural method of genetic exchange, carries the code for self-regulation and evolutionary adaptation. With the rapid advancement of gene editing technologies, the unique advantages of natural transformation are being increasingly recognized and applied in this cutting-edge field. This chapter will analyze the practical applications of natural transformation in gene editing and highlight its recent discoveries in the field.

### 5.1 Gene editing of difficult-to-edit strains

Natural transformation is widely used for gene editing in synthetic biology, especially for strains that are difficult to manipulate (Kearns and Losick, 2003), In wild-type strains isolated from the environment, conventional genetic modification methods—such as phage transduction, electroporation, and protoplast transformation—are often hindered by high host specificity, technical complexity, and low chromosomal integration efficiency. Recent studies have shown that these barriers can be overcome by artificially regulating key regulatory genes involved in natural transformation (Romero et al., 2006; Masood et al., 2011). Nijland et al. (2010) showed that overexpressing the comK gene, which regulates natural transformation in *Bacillus subtilis*, enhanced strains’ transformation efficiency and facilitated gene editing of wild-type strains. Mulder et al. (2017) overexpressed the *comX* gene in the lactic acid bacterium *KF147*, leading to a remarkable 4,000-fold increase in gene editing efficiency. This approach has also been successfully used in clinically important, genetically intractable strains, such as *Streptococcus pyogenes*. By optimizing competence induction conditions, it allows the efficient generation of multi-site gene deletion mutants (Mårli et al., 2024).

### 5.2 Targeted chromosomal mutagenesis

Natural transformation is an efficient gene editing tool that offers significant advantages in targeted chromosomal mutagenesis. Its core mechanism relies on the bacterium’s natural competence to actively take up exogenous DNA, which enables precise gene modification through homologous recombination. When the exogenous DNA fragment shares high homology with the target gene regions on the host strain’s chromosome, the cell’s recombinase system, such as RecA protein (De Lemos et al., 2025), recognizes these homologous sequences. It then mediates DNA strand breakage, exchange, and rejoining, thereby integrating the exogenous DNA precisely into the targeted chromosomal location for gene editing. The process of targeted chromosomal mutagenesis in the natural transformation system is both rigorous and efficient. Nishikawa and Tanaka (2013) applied natural transformation to perform targeted chromosomal mutagenesis in *Fusobacterium nucleatum*. By utilizing natural competence, they successfully constructed gene-deficient strains. The method involved a recombinant plasmid, constructed in *Escherichia coli*, which inserted an antibiotic resistance gene into the target gene. Subsequently, a specialized microenvironment created by the biofilm induces *Fusobacterium nucleatum* to enter a naturally competent state, enabling them to actively take up exogenous DNA. When recombinant plasmids are introduced into the environment containing competent bacteria, DNA-binding proteins and transport systems on the bacterial surface quickly recognize and take up the recombinant plasmids. The cell’s recombinase system then facilitates two precise exchanges between the recombinant plasmid and chromosomal DNA, integrating the target gene fragment carrying the resistance gene into the chromosome. This process replaces the original wild-type gene, resulting in a targeted chromosomal mutation. Natural transformation significantly improved the efficiency of single-point mutations, offering advantages over traditional broth culture and electroporation methods, including cost-effectiveness and high reproducibility (Nishikawa and Tanaka, 2013).

Compared to random insertion gene editing methods, targeted chromosomal mutagenesis provides distinct advantages. By utilizing the highly conserved mechanism of homologous recombination, it ensures the precise integration of exogenous DNA fragments into the chromosome, avoiding issues such as gene dysfunction and chromosomal instability caused by random insertions. This substantially enhances the accuracy of gene editing (Huck et al., 2019). With these advantages, targeted chromosomal mutagenesis shows significant potential for application across multiple fields. In basic research, scientists can efficiently construct gene-deficient strain libraries to analyze gene functions and regulatory networks systematically. In synthetic biology, precise editing of industrial strain chromosomes can optimize metabolic pathways, greatly enhancing the synthesis of high-value products such as biofuels and pharmaceutical precursors. In medical research, targeted mutagenesis of pathogenic bacteria helps uncover pathogenic mechanisms, providing crucial insights for developing novel antimicrobial strategies. As the technology continues to improve, targeted chromosomal mutagenesis is expected to achieve breakthroughs in strains that are difficult to transform.

### 5.3 Multiplex genome editing

Editing bacterial genomes is a crucial tool in synthetic biology research and applications. While methods for generating single defined mutations in bacterial genomes have been developed, generating multiple mutations simultaneously (multiplex genome editing) remains limited. In bacterial genome editing, multiplex genome editing techniques have opened new avenues for analyzing complex gene networks and optimizing strain performance. Among these, natural transformation-mediated multiplex genome editing provides distinct advantages. Previously, multiplex genome editing mainly involved CRISPR/Cas-based techniques (Sander and Joung, 2014) and Multiplex Automated Genome Engineering (MAGE) technology (Wang et al., 2012). Although these methods have shown success in microbial systems, they are primarily applicable to model bacteria like *Escherichia coli*. Multiplex natural transformation genome editing in bacteria is a gene editing technology that utilizes the bacterium’s natural transformation ability, enabling the introduction of multiple gene mutations in a single transformation, thus significantly increasing transformation efficiency. The core principle relies on the bacterium’s ability to actively uptake free extracellular DNA and integrate it into its genome via homologous recombination. This process does not require external protein assistance, but two conditions must be met: first, the DNA fragments to be edited must have homologous sequences longer than 2000 bp on both sides to promote efficient homologous recombination. For example, in *Vibrio* species, short homologous arms lead to the degradation of exogenous DNA and failed transformation. Second, bacteria must be capable of simultaneously taking up multiple linear DNA fragments, each carrying different editing sites, to achieve multi-site editing. This is exemplified by *Campylobacter jejuni*, which exhibits high co-transformation efficiency and can introduce multiple mutations in a single transformation (Yamamoto et al., 2021). Dalia et al. (2014) described a multiplex genome editing approach using natural transformation, demonstrating its effectiveness in *Vibrio cholerae* and *Streptococcus pneumoniae*. Their study revealed an unprecedented 50% genome editing frequency and highlighted the method’s utility in directed evolution studies (Dalia et al., 2014). Dalia et al. (2017) used multiplex genome editing to target nine genes involved in poly-β-hydroxybutyrate (PHB) biosynthesis in *Vibrio natronobacterium*, resulting in a 100-fold increase in PHB production (Dalia et al., 2017). Yu et al. (2019) used MuGENT to construct five gene mutants in *Vibrio vulnificus*, successfully reducing strain virulence by 668-fold. Glasgo et al. (2024) demonstrated that MuGENT could be used for gene complementation via insertion at ectopic chromosomal sites. They also demonstrated MuGENT’s powerful gene deletion capability, creating a 280 kb deletion in a single round of bacterial targeted mutagenesis—one of the largest artificial deletions ever achieved (Glasgo et al., 2024).

Multiplex gene editing and regulation technologies using the CRISPR/Cas system represent significant advances in genetic engineering. These technologies primarily target multiple gene loci simultaneously or regulate the expression of several genes to achieve complex genome modifications and network control. In multiplex gene editing, multiple gRNA arrays are constructed, utilizing endogenous RNases (such as the RNase activity of Cas12a) or exogenous processing elements (e.g., tRNA or ribozymes) to cleave long-chain RNA and generate mature gRNAs (McCarty et al., 2020; Lim and Lee, 2024). For example, the CRISPR/Cas9 system can express 4–8 gRNAs from a single vector to achieve multi-gene knockouts or large fragment deletions. Iterative editing systems based on CRISPR/Cas nucleases reduce off-target effects through a stepwise approach, enabling multiple rounds of editing in bacteria with single-cycle efficiencies exceeding 80% (Liu et al., 2019). The CRISPR-Combo platform, which combines Cas9 with transcriptional activation domains (e.g., VP64), enables simultaneous gene knockout and activation within the same system, as demonstrated in plant breeding applications (Pan and Qi, 2023). Compact Cas proteins, such as Cas12f (Un1Cas12f1), are more easily delivered through viral vectors due to their small size (529 amino acids), supporting multiplex editing (Park et al., 2019). In multiplex gene regulation, inactivated dCas9 or ddCpf1 fused with effector domains (e.g., the KRAB repressor or p65 activator) can achieve coordinated regulation of multiple genes. For example, the ddCpf1 system uses a single crRNA array to regulate the expression of five two-component systems in *Escherichia coli* for screening key metabolic pathway targets (Zhang et al., 2017). In metabolic engineering, multiplex gene editing technologies enable simultaneous regulation of multiple glycolytic genes and cofactor synthesis genes in *E. coli*, increasing butanol production threefold. In synthetic biology, these technologies can rapidly re-engineer multiple metabolic nodes in a single step, reconstructing complex metabolic pathways for the efficient synthesis of high-value products. In basic research, these technologies help elucidate the synergistic networks between genes, providing key tools for studying multi-gene regulatory mechanisms.

### 5.4 Marker-free genome editing

As mentioned earlier, natural transformation-based targeted chromosomal mutagenesis still relies on the classic natural genetic transformation method for genome editing. The selective markers used in this approach can affect gene expression, potentially leading to phenotypic effects, and may accumulate as unwanted genes in subsequent cycles of mutagenesis. Marker-free gene editing technology effectively avoids the potential interference of traditional antibiotic resistance markers through innovative strategies. Its core mechanism relies on the synergistic action of the bacterium’s natural genetic transformation process and the CRISPR-Cas9 counter-selection system. In *Streptococcus thermophilus* and *Streptococcus pneumoniae*, researchers have optimized the efficiency of natural transformation and designed linear donor DNA with homologous flanking regions to achieve precise editing of targeted chromosomal loci. The breakthrough of this method lies in its complete reliance on the bacterium’s intrinsic DNA uptake and homologous recombination capabilities, without the need for exogenous plasmid vectors or antibiotic selection markers, thus avoiding the potential effects of residual foreign sequences on gene expression (Stukenberg et al., 2022). Junges et al. (2023) proposed a method for efficient, precise marker-free genome editing by enhancing natural transformation efficiency in strains. This method combines specific induction peptides with donor amplification products containing extensive flanking homology. It leverages natural transformation’s ability to accurately bind, uptake, transport, and recombine foreign DNA fragments, enabling marker-free gene editing without including unnecessary DNA vector sequences. Using this technique, specific base pair deletions and substitutions have been successfully made in *Streptococcus pneumoniae* and *Streptococcus pyogenes*. Bailo et al. (2019) introduced marker-free genome editing in *Legionella pneumophila*. They used the natural competence of *Legionella pneumophila* and linear DNA fragments as recombinant substrates, eliminating the need for an intermediate host to amplify the target DNA (Bailo et al., 2019). Based on a suicide box strategy, this genetic toolbox enables marker-free gene editing and insertion of sequences at selected locations on the *Legionella pneumophila* chromosome, enabling multiplex genome editing.

Stukenberg et al. (2022) described a method combining natural transformation with CRISPR-Cas9-based counter-selection for marker-free genome editing in bacteria, termed NT-CRISPR.

By combining natural transformation with the counter-selection capability of CRISPR-Cas9, a two-step editing process is established: donor DNA carrying the target mutation is first introduced into the cell via natural transformation, where it undergoes homologous recombination with the genome, resulting in a population of edited and unedited cells. Next, sgRNAs are designed to specifically target the original wild-type sequence, and the Cas9 protein induces lethal DNA double-strand breaks in cells that have not been successfully edited, while cells with successful edits are protected from cleavage due to changes in the target sequence, achieving an editing efficiency of 99.999%. This strategy has enabled single-base substitutions (e.g., C→T transition) and gene deletions up to 500 bp in *Streptococcus thermophilus*, without the need for antibiotic selection. Through iterative application, multiple gene loci can be edited in the same strain. For instance, in *Streptococcus pneumoniae*, researchers completed gene deletion, point mutation, and exogenous gene integration over three independent editing rounds, with each round eliminating residual markers through CRISPR-Cas9 counter-selection, generating triple mutant strains free of any exogenous sequences. Furthermore, this technology is applicable to large fragment deletions and multiplex genome editing by designing composite donor DNAs containing multiple mutation sites, combined with multi-sgRNA arrays for efficient screening. Compared to traditional methods, NT-CRISPR technology presents three major breakthroughs: first, it eliminates phenotypic interference, avoiding unintended effects of resistance markers on bacterial metabolic pathways, virulence factors, or competitive fitness; second, it improves editing precision, increasing efficiency from less than 1% in traditional methods to over 90%, with off-target rates below detection limits. This technology provides more precise and safer genetic tools for bacterial functional genomics, industrial strain optimization, and pathogenicity mechanism analysis (Haapaniemi et al., 2018; Stukenberg et al., 2022).

In addition to the previously mentioned natural transformation applications, some modified methods enhance natural transformation—through chemical, physical, and electrical processes, as well as other techniques—by permeabilizing bacterial cell membranes to allow plasmid DNA uptake. Methods such as electroporation and chemical transformation also leverage the molecular mechanisms of natural transformation in bacterial strains and are widely used in laboratory settings (Ren et al., 2019).

## 6 Conclusion and outlook

Although natural transformation has been studied for nearly a century, many aspects of the molecular mechanisms underlying natural transformation in various bacterial strains remain unexplored. Furthermore, previously studied genes may play additional roles in natural transformation. For example, in the Gram-positive bacteria discussed earlier, the ComGC pilus is reported to have two distinct structural forms. Laurenceau et al. (2013) proposed that ComGC can form long type IV pilus structures, measuring 2–3 μM in length and 5 nm in width, extending from the bacterial surface. The authors confirmed this filamentous pilus structure using immunogold labeling and mass spectrometry (Laurenceau et al., 2013). In contrast, Balaban et al. (2014) observed short, distinct pilus structures composed of ComGC using electron microscopy, which were absent in ComGC mutant strains. These structures had a diameter of 8–10 nm and an average length of approximately 100 nm (Balaban et al., 2014). Additionally, two DNA uptake models—retraction or capture models and cell wall pore models—have been proposed based on these observations (Muschiol et al., 2015). Therefore, exploring the mechanisms of natural transformation requires further research to better understand its role in the natural transformation of industrially important strains. The biological functions of natural transformation remain unclear. This article summarizes several hypotheses, including the roles of natural transformation in genome evolution, environmental adaptation, genome repair, and biofilm formation. Describing the biological functions of natural transformation will contribute to a more thorough understanding of its significance in bacterial strains.

Of course, the most important aspect is the practical application of natural transformation. This article systematically reviews the gene editing technologies developed through natural transformation in recent years. Natural transformation presents numerous possibilities for practical applications. As our understanding of natural transformation mechanisms continues to advance and gene editing technologies evolve, natural transformation is expected to play an increasingly important role across a wide range of fields.
